# Peptide-Based Inhibitors of Protein–Protein Interactions (PPIs): A Case Study on the Interaction Between SARS-CoV-2 Spike Protein and Human Angiotensin-Converting Enzyme 2 (hACE2)

**DOI:** 10.3390/biomedicines12102361

**Published:** 2024-10-16

**Authors:** Aizhan Rakhmetullina, Piotr Zielenkiewicz, Norbert Odolczyk

**Affiliations:** 1 Institute of Biochemistry and Biophysics, Polish Academy of Sciences, 02-106 Warsaw, Poland; arakhmet@ibb.waw.pl (A.R.); piotr@ibb.waw.pl (P.Z.); 2 Department of Systems Biology, Institute of Experimental Plant Biology and Biotechnology, University of Warsaw, Miecznikowa 1, 02-096 Warsaw, Poland

**Keywords:** SARS-CoV-2, COVID-19, inhibitors of protein–protein interactions, peptides, drug design, coronavirus, angiotensin-converting enzyme 2, ACE2

## Abstract

Protein–protein interactions (PPIs) are fundamental to many critical biological processes and are crucial in mediating essential cellular functions across diverse organisms, including bacteria, parasites, and viruses. A notable example is the interaction between the SARS-CoV-2 spike (S) protein and the human angiotensin-converting enzyme 2 (hACE2), which initiates a series of events leading to viral replication. Interrupting this interaction offers a promising strategy for blocking or significantly reducing infection, highlighting its potential as a target for anti-SARS-CoV-2 therapies. This review focuses on the hACE2 and SARS-CoV-2 spike protein interaction, exemplifying the latest advancements in peptide-based strategies for developing PPI inhibitors. We discuss various approaches for creating peptide-based inhibitors that target this critical interaction, aiming to provide potential treatments for COVID-19.

## 1. Introduction

For many decades, peptides were underrated as therapeutic agents due to inherent challenges such as high molecular weight, poor solubility, membrane impermeability, and biological instability [1]. However, the development of peptide-based drugs has recently become a prominent topic in the pharmaceutical industry and in scientific research [2]. As essential functional molecules in nature, peptides are well suited to interact with biological targets, leading to their resurgence as valuable building blocks in medicinal chemistry and promising candidates for drug discovery. Peptides possess unique features that are often unattainable by small molecules, such as a high specificity, strong efficacy, low toxicity, and the ability to target larger surface areas, including protein receptors and protein–protein interaction (PPI) interfaces [3,4,5,6,7].

PPIs are involved in many critical biological processes in living organisms and play a crucial role in mediating many important cellular functions, including vital biosynthetic pathways in bacteria, parasites, and viruses [8]. A subset of pathogen PPIs, which are essential for pathogenesis, engage in competitive binding with host proteins—a phenomenon known as cross-species interactions [9]. By mimicking the interface between host proteins, pathogens can hijack critical cellular functions for their own benefit, such as blocking the host’s immune response or facilitating viral entry into cells [10].

Paradoxically, one well-studied system is the interaction between the SARS-CoV-2 spike protein (S) and its human protein receptor, angiotensin-converting enzyme 2 (*h*ACE2), which was only recently discovered and characterized during the outbreak of coronavirus disease (COVID-19) [11]. The interaction between the S protein and the *h*ACE2 receptor triggers a cascade of events that ultimately lead to viral replication [12]. Disrupting the recognition process of the *h*ACE2 receptor by the S protein could block or significantly reduce infection by the novel pathogen, making it a promising target for potential anti-SARS-CoV-2 therapies. Thus, many studies have focused on identifying small molecules that target the S protein or the *h*ACE2 receptor to disrupt their complex formation and potentially prevent SARS-CoV-2 infection [13,14,15]. An approach that uses peptides to inhibit this crucial recognition process, which is essential for viral cell penetration, may offer an innovative treatment option to prevent the spread of the SARS-CoV-2 virus.

We chose the interaction between *h*ACE2 and the SARS-CoV-2 spike protein as an exemplary case of current, state-of-the-art advancements in peptide-based strategies for designing and developing protein–protein interaction inhibitors. In this review, we also summarize the various approaches to developing peptide-based inhibitors, targeting the *h*ACE2 and S interaction as potential treatments for COVID-19. To examine previously reported therapeutic peptides as inhibitors of S-*h*ACE2 interaction, we searched PubMed using the keywords “peptides as inhibitors of SARS-CoV-2”. From the results, we focused exclusively on studies that experimentally demonstrated the ability of these peptides to block the interaction or to interact with one of the protein partners involved in the complex formation. The studies we found were divided into two groups: the first group contained native peptides, while the second group consisted of optimized peptides, primarily modified by natural amino acid residues.

## 2. Complex Structure of *h*ACE and Spike Proteins

The SARS-CoV-2 contains envelope (E), membrane (M), nucleocapsid (N), and spike (S) structural proteins [16,17]. The latter, which is crucial for the virus’s entry into host cells, is composed of two subunits, S1 and S2. The S1 subunit includes the receptor-binding domain (RBD) that specifically attaches to the *h*ACE2 receptor on host cells, primarily epithelial cells in the respiratory tract. The S2 subunit facilitates the fusion of the viral membrane with the host–cell membrane, a critical step for viral entry [18,19]. The S protein by RBD recognizes the extracellular peptidase domain (PD) of the *h*ACE2 receptor of host epithelial cells, which initiates infection by virus attachment and its entry into the cells [11,20,21].

**Figure 1:**
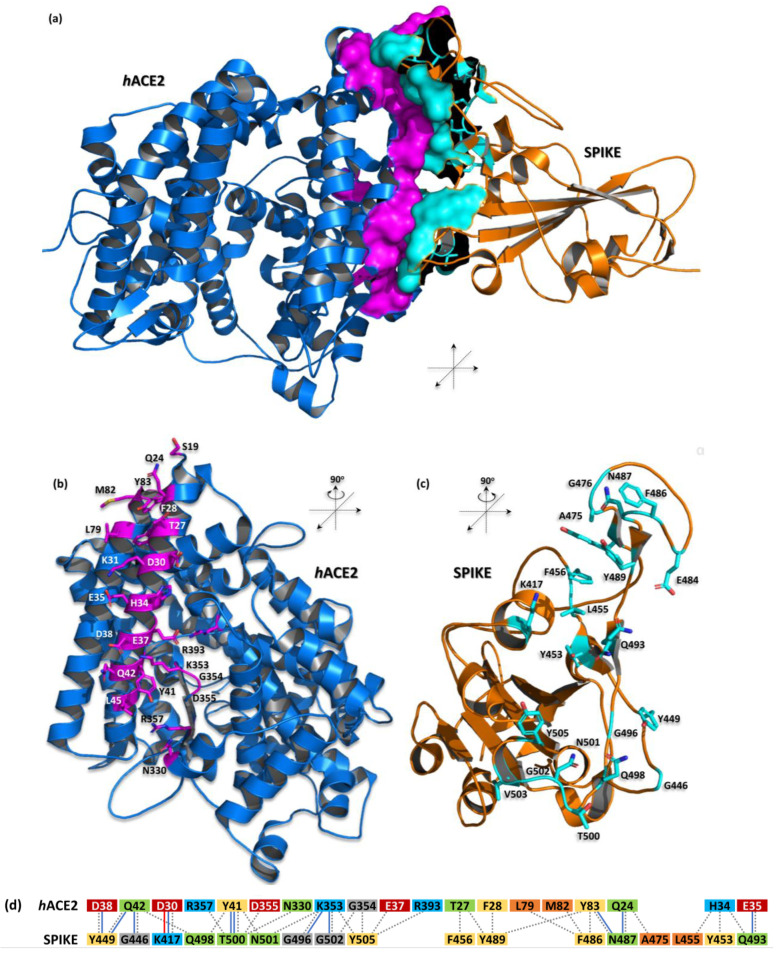
The complex structure of *h*ACE2 and SARS-CoV-2 spike proteins (PDB id: 6m0j)— computer-generated, cartoon representation. (**a**) The interaction interface between *h*ACE2 (blue) and the spike (orange) is shown as the solvent accessible surface area and highlighted by magenta and cyan colors, respectively; (**b**,**c**) depicted amino acid residues forming the interface for a particular protein are shown as sticks in this representation; (**d**) schematic diagram of interactions between proteins. Residues are colored according to the type: positive (H, K, R); negative (D, E); S, T, N, Q = neutral; A, V, L, I, M = aliphatic; F, Y, W = aromatic; G = Gly. Type of contacts: hydrogen bonds (blue line); salt bridges (red line); nonbonded contacts (gray dash line). Protein visualization was prepared by the PyMOL Molecular Graphics System, Version 3.0.0 Schrödinger, LLC.

The structure of *h*ACE2 and the spike of the SARS-CoV-2 (RBD) proteins complex were first proposed by homology modeling [22]; these have since been supported by experimental techniques such as cryo-electron microscopy [23] and X-ray diffraction crystallography [11,21,23,24]. The SARS-CoV-2 spike RBD has a significant interaction interface with the N-terminal α1 helix (residues S19-T52) of *h*ACE2. This fragment engages with the outer surface of the viral receptor-binding domain (RBD) and features a substantial buried surface area of 1688 Å^2^. This area is composed of 19 residues from *h*ACE2 (825 Å^2^) and 17 residues from the viral protein (863 Å^2^) [11] (Table 1/Figure 1). The interaction is predominantly polar, involving 11 hydrogen bonds and one salt bridge between the K417 of the RBD and the D38 of *h*ACE2 (Table 1/Figure 1). These interactions stabilize the complex, resulting in an equilibrium dissociation constant (K_D_) of approximately 14.7 nM [25].

## 3. Native Peptides

Several studies have demonstrated that peptides derived directly from the native sequence of one of the interacting partners serve as valuable starting points for developing effective PPI inhibitors [26,27,28,29]. Since the structure of the *h*ACE2 and SARS-CoV-2 spike protein complex has been known since the early days of the pandemic [23], efforts to find peptides that effectively block this interaction began immediately after the first COVID-19 cases, employing various approaches including small molecules screening, peptide design, the production of monoclonal antibodies, and chimeric proteins.

However, it is important to note that the concept of blocking the interaction between the *h*ACE2 receptor and viral protein was not new, even within the field of coronaviruses. The initial studies demonstrating this antiviral strategy were published by Ho and colleagues nearly two decades before first COVID-19 case was detected in humans [30]. Their research showed that small peptides derived from the viral S protein of SARS-CoV could effectively block the interaction between *h*ACE2 and the S protein of the closely related virus to SARS-CoV-2. Out of the 14 peptides tested, 4 were identified as active and successfully inhibited the *h*ACE2-S interactions, with a half maximal inhibitory concentration (IC_50_) in the low nanomolar range [30]. This foundational work provided a basis for similar approaches during the COVID-19 pandemic.

Following the identification of SARS-CoV-2 [31,32], the first research papers demonstrating the efficacy of native fragment peptides emerged a few months later [33]. This initial research paved the way for a prolific output of studies on peptides and peptide-derived molecules, which have shown considerable therapeutic potential, as documented extensively in the scientific literature.

The knowledge about the structure of complex SARS-CoV-2 and *h*ACE2 proteins, along with the experience gained with SARS-CoV [34], initiated studies which resulted in a number of publications that demonstrated promising outcomes on the use of peptide inhibitors; these were designed by combining the critical residues in the protein interface to prevent the entrance of SARS-CoV-2.

One of the first studies that experimentally validated peptides that may block the interaction between *h*ACE2 and the S protein was conducted by Larue and colleagues [33]. Their study involved a rational analysis of the interacting interfaces of both proteins, focusing primarily on the 27-TFLDKFNHEAEDLFYQ-42 fragment of the N-terminal α1 helix of the *h*ACE2 receptor. They proposed four native sequence fragments from α1 for peptide synthesis: **SAP1**, **SAP2**, **SAP5**, and **SAP6**, containing 16, 9, 13, and 6 residues, respectively (Table 2). Additionally, they investigated two peripheral regions of the *h*ACE2 interface, resulting in peptides **SAP3** and **SAP4**, which contained 7 residues from α3 and 8 residues from α11, respectively. The synthetic peptides **SAP1** (16-mer) and **SAP6** (6-mer) have similar binding affinities to the viral protein, with K_D_ values of 0.53 ± 0.01 mM and 1.36 ± 0.14 mM, respectively.

Based on the luciferase assay system results, they revealed that the inhibitory effects of the *h*ACE2-derived peptides on pseudovirus infection were comparable, with IC_50_ values of 2.39 mM for **SAP1** and 1.90 mM for **SAP6**. In contrast, **SAP3**, **SAP4**, and **SAP5** were identified as poor or non-inhibitors of spike-mediated virus infection. According to their findings, the minimum number of residues required for the effective inhibition of SARS-CoV-2 was found in the 6-mer peptide **SAP6** (37-EDLFYQ-42) [33].

Another research group, as presented by Pei et al., investigated short native sequence peptides derived from the α1-helix of *h*ACE2 [35]. They designed the octapeptide **SI4α** and the heptapeptide **SI5α**. Notably, these peptides did not include the crucial binding sequence to the spike protein identified by Larue [33]. Specifically, **SI4α** consists of residues Q24-K31, while **SI5α** is a shortened version ending before K31. Consequently, both peptides partially overlap with the sequence of **SAP5**, which was identified as a poor inhibitor by Larue [33].

Despite this, Pei et al. found that **SI5α** was one of the shortest native peptides with significant antiviral activity, demonstrating a half maximal effective concentration (EC_50_) value of 1.59 μM against the model coronavirus GX_P2V. Additionally, **SI5α** inhibited the interaction between the RBD and *h*ACE2 by approximately 75% and 81% at concentrations of 50 µM and 100 µM, respectively [35].

Unlike the studies by Larue and Pei, Abouhajar et al. focused on synthesizing and testing slightly longer *h*ACE2-derived peptides aimed at disrupting the ACE2/RBD complex [36]. They designed 23-mer and 13-mer peptides based on analyses of ACE2/SARS-CoV-2 S-protein interaction residues from prior ACE2/RBD crystal structure studies. Their objective was to evaluate how variations in peptide length affect the blocking efficiency of the SARS-CoV-2 spike protein. The researchers created two potential peptide inhibitors: **Peptide** [22,23,24,25,26,27,28,29,30,31,32,33,34,35,36,37,38,39,40,41,42,43,44] and **Peptide** [30,31,32,33,34,35,36,37,38,39,40,41,42], designed to bind to the SARS-CoV-2 S protein and disrupt its interaction with the human ACE2 receptor. The binding of these peptides to the spike protein was assessed using a surface plasmon resonance (SPR) competitive assay. The results showed that increasing the concentration of both peptides reduced the binding signal of the *h*ACE2/RBD complex. Interestingly, the 13-mer peptide (D30–Q42), a truncated version of the 23-mer peptide (E22–S44), exhibited more effective inhibition, with an IC_50_ value of 0.65 μg/mL, compared to the 23-mer peptide, which had an IC_50_ of 2.00 μg/mL. This suggests that longer peptides do not always enhance binding affinity toward the S protein [36].

Another study also focused on the *h*ACE2 α1 helix and identified one of the longest peptides, **Pep 1,** containing 27 native residues (S19–L45) from a human receptor, which was further used for the optimization process (see next chapter) [37].

## 4. Mutated Peptides

The studies presented above demonstrate that carefully selected and extracted fragments of protein–protein interaction interfaces can play a crucial role in developing peptides that effectively block unwanted complex formation. The thorough analysis of the 3D structure of these complexes, including the types of amino acid residues and the complementarity between interacting surfaces based on their physicochemical, geometrical, and distance-related properties, can provide valuable insights for designing such peptides. However, peptide fragments extracted from the protein context often exhibit increased flexibility and may face challenges in becoming effective drug candidates. Therefore, these peptides typically require further optimization. This optimization is usually a multistep, iterative process that results in various derivatives. These derivatives may or may not possess improved properties, which can enhance their efficacy as inhibitors of protein–protein interactions and increase their therapeutic potential.

The peptides optimization process may be oriented on different aspects of such molecules like (1) blocking their degradation/improving their stability in the organism, (2) increasing their solubility, (3) ameliorating cell membrane penetration, or (4) improving some ADMET (Absorption, Distribution, Metabolism, Excretion, and Toxicity) properties, just to name a few. In this paper, we would like to highlight the methods of optimization which solely modify their sequences, leading to new optimized molecules which chemically remain pure protein amino acid peptides (with a few exceptions that are standard procedure in peptide synthesis chemistry like capping N- and C- terminals). More sophisticated chemical modifications, such as adding functional groups or stapling peptide structures, which involve complex alterations, are beyond the scope of this review.

One of the first optimized peptides targeting the recognition of the *h*ACE2 receptor by the SARS-CoV-2 RBD spike protein was proposed by Karoyan et al. [38]. They started with the N-terminal α1 helix of *h*ACE2 (19S-L45) as the base sequence for further optimization (Table 3). Their approach aimed to achieve two main objectives through peptide modifications. First, the optimized peptides needed to maintain comparable binding affinity to the spike RBD as the native *h*ACE2, while also exhibiting low antigenicity to avoid triggering a neutralizing immune response. This was accomplished by preserving crucial residues from the native *h*ACE2 that are responsible for binding, while substituting non-essential residues with Ala and/or Leu, which have higher helical folding propensities. This strategy aimed to reduce the entropy penalty upon peptide binding. The second goal was to minimize antigenicity. This was achieved using a semi-empirical method [39] to identify potential antigenic determinants, followed by the iterative testing of each new sequence. Among the ten different 27-mer and 29-mer peptides tested, three peptides—**P8**, **P9**, and **P10**—were found to inhibit SARS-CoV-2 effectively, with inhibitory concentrations in the nanomolar range and high helical folding propensities ranging from 53% to 70%.

Among the peptides designed to target ACE2, **P10** emerged as one of the most promising. It was demonstrated to block SARS-CoV-2 infection in human pulmonary cells, with a binding affinity of ~0.03 nM as measured by biolayer interferometry and an IC_50_ value of ~42 nM as assessed by ELISA assays. Notably, the peptides **P8**, **P9**, and **P10** exhibited no cytotoxicity in Vero-E5 and Calu-3 cell cultures [38].

Chitsike et al. focused on the same fragment of the *h*ACE2 N-terminal α1 helix (S19-L45) as Karoyan et al., proposing various modified peptides based on this native sequence [37]. They selected this fragment based on computational alanine-scanning mutagenesis (using Rosetta and Bude), which identified S19, Q24, T27, F28, E30, K31, H34, E35, E37, D38, Y41, and L45 as key residues with a significant impact on binding energy. **Pep 1**, as described above, was directly adopted from the native sequence, whereas **Pep 2** was developed by combining the part of the **Pep 1** sequence (24–45) with an additional fragment of the *h*ACE2 interface, residues 351-LGKGDFR-360, linked via the glycine residue to the C-terminal of **Pep1**. For the next three peptide variants, the original sequence was truncated at the N-terminal, and new mutations were introduced to enhance binding affinity and/or improve structural stability in the solution. Thus, **Pep 3** consisted of *h*ACE2 residues Q24-L45 with four mutations (A25V, T27Y, L39R, F40D) which were found to enhance binding affinity by other studies [40]. **Pep 4** and **Pep 5** were designed based on the T27-A46 fragment, incorporating several mutations to enhance helical folding by introducing an amphipathic α-helical pattern, with both peptides also having their N-terminal phenylalanine residues replaced with proline to reduce the likelihood of proteolytic degradation. Additionally, the **Pep 5** includes a covalent side-chain cyclization designed to stabilize the α-helical structure. **Pep 6**, on the other hand, was derived from the S1 RBD fragment (483V–505Y), which is critical for host receptor recognition during viral attachment. An AlphaScreen™ assay was used to evaluate the ability of these peptides, both with and without modifications, to block ACE2-RBD binding. Notably, the SARS-CoV-2 RBD-derived 23-mer **Pep 6** exhibited the highest activity, with an IC_50_ of 27 μM, outperforming most of ACE2-derived peptides, which had IC_50_ values ranging from 42 μM to 363 μM [37].

Chopra and colleagues initially identified a 24-mer fragment of *h*ACE2 (S19-Q42) that encompasses most of the known contact residues. They discovered that shifting this sequence of nine residues toward the N-terminal direction of *h*ACE2 protein increased the binding affinity to the SARS-CoV-2 RBD [41]. Following this, they systematically permuted the shifted *h*ACE2 fragment (S10–N33) to identify peptides with higher binding affinities to the viral protein. Ultimately, they identified 22 peptides with a three-fold greater binding affinity compared to the initially proposed shifted peptide. Among these, only one peptide, **BP19**, effectively inhibited the interaction between the viral protein and the human receptor, with an IC_50_ of 2.08 ± 0.38 μM, as measured by a competitive ELISA assay for *h*ACE2 protein interaction. **BP19** competed with approximately 13.48 μM of *h*ACE2. Notably, all mutations except one (N33I) were introduced in the shifted 9-residue N-terminal fragment, below the serine at position 19.

Rajpoot et al. initiated their peptide development using a slightly shorter 13-mer fragment of the *h*ACE2 α1 helix, starting from residue F28. They performed mutation studies to enhance binding affinity. Their research highlighted the importance of substituting specific amino acid residues (E8N, A9F, and E10K) to increase binding affinity without disrupting the RBD binding site or adversely affecting the peptide’s physicochemical properties. Among the peptides tested, the **13AApi** peptide demonstrated the ability to inhibit the interaction between the SARS-CoV-2 spike protein and *h*ACE2, achieving a minimal inhibition of 40% at a 100 µM concentration, as assessed by ELISA [42].

One of the shorter peptides were proposed by Odolczyk et al. [27]. Based on their findings that the interaction between *h*ACE2 and RBD is primarily mediated by the D30-Q42 fragment [27], they designed three short peptides: **pep1c**, **pep1d**, and **pep1e**, each incorporating a single mutation compared to the native *h*ACE2 sequence. The rationale behind these mutations was to prevent the aggregation of the peptide fragments with the *h*ACE2 protein during its folding process, which could be an undesired effect for potential future drug molecules. Among the peptides developed, the 9-mer **pep1c** and the 6-mer **pep1d** both bound to the spike protein with similar affinities (K_D_ ~280 nM and ~210 nM, respectively). However, only **pep1c** was tested for its ability to inhibit the RBD-*h*ACE2 complex formation, achieving an IC_50_ of approximately 3.3 mM.

In the continuation of their studies, Odolczyk et al. applied in silico modeling to optimize the sequence of **pep1d**, aiming to enhance peptide–protein interactions and increase binding affinity to the spike protein [43]. The in silico analysis, complemented by microscale thermophoresis (MST) and ELISA assays, led to the development of an *h*ACE2-based peptide named **J3**. The **J3** peptide, with the sequence DYGNHE, showed a significantly improved binding affinity (K_D_ ~50 nM) compared to the previous peptides. This enhancement was achieved by substituting the positively charged Lys at the second position of the 6-mer peptide with the neutral hydrophobic Tyr [43]. The **J3** peptide effectively diminished the *h*ACE2–spike interaction, with the measured K_D_ changing from ~151 nM in the absence of the peptide to ~720 nM in the presence of 1 mM of peptide. The experimental data of peptides activity verification were summarized in Table 4.

## 5. Discussion

SARS-CoV-2 is responsible for the acute respiratory infection that has caused large numbers of cases and deaths worldwide. For a long time, there was no specific therapy for COVID-19 disease; this created an urgent need to develop a new drug against the virus [44,45]. However, the traditional drug discovery process takes a long time, which is not suitable for the fast reaction that is needed for new emerging threats caused by rapidly spreading pathogens.

Research has consistently shown that peptides derived directly from the native sequences of interacting partners within protein–protein interaction (PPI) interfaces provide an effective foundation for developing protein–protein interaction inhibitors [26,27,28,29]. These peptides are particularly valuable because they mimic the natural interaction sites and may offer a targeted approach to modulate biological pathways involved in diseases.

Therapeutic peptides are new and promising alternatives to treat numerous diseases, including cancer, metabolic disorders, infectious, and cardiovascular and neurodegenerative diseases [2,46], including COVID-19 [47,48,49,50,51,52]. The features that make peptides suitable for therapeutic candidates include their accessibility, structural and functional diversity, high specificity and affinity in low concentrations, high level of biological activity, low production costs, low immunogenicity, and ease of synthesis and storage [3,4,5,6,7]. They are small in size and do not accumulate in specific organs, which helps to reduce their toxic side effects.

Another advantage of antiviral peptides is that in many cases they are designed to act extracellularly and do not need to pass the cell membrane, reducing the possibility of virus–host cell interactions [2]. Because of their simplicity of synthesis, peptide sequences can also be easily modified via chemical or molecular biology approaches, for example, to react in response to emerging new drug-resistant strains of a virus.

However, peptides also encounter several challenges related to their direct applications as drug molecules. Thus, it is crucial to address to main limitations associated with their use in living organisms and to explore potential strategies to overcome these obstacles. One of the main challenges with peptide drug molecules is their poor stability, as they are highly susceptible to degradation by proteolytic enzymes. This results in a short half-life and reduced efficacy, limiting their therapeutic potential.

To address this issue, peptide stapling has emerged as a promising approach. This technique involves incorporating lactam-based i and i + 4 linkages between amino acids to stabilize the peptide’s structure, thereby enhancing its resistance to proteases. For example, the stapled peptide hACE2_21-55 (A36K-F40E) has demonstrated an effective inhibition of the SARS-CoV-2 spike protein binding to *h*ACE2, with promising in vitro results (IC_50_ = 3.6 μM, K_D_ = 2.1 μM) [53]. Introducing such constraints may also lock the peptide molecule in a bioactive conformation, resulting in more efficient interactions with its target. However, it is worth mentioning that this approach may also reduce binding affinity when the target recognizes the unstructured peptide. In such cases, stapled peptides may have fewer opportunities to bind, which could negatively impact their binding affinity [54].

Proteolysis often initiates at the N- or C-termini due to the action of various proteases and peptidases. Different amino acid residues at the termini exhibit varying degrees of resistance to degradation, with some being more susceptible to proteolysis [55]. For instance, N-terminal residues like Met, Ser, Ala, and Gly tend to enhance stability, while sequences rich in Pro, Glu, or Thr are more prone to degradation. By modifying the terminal sequences without compromising the peptide’s targeting or binding properties, degradation can be minimized, leading to enhanced bioavailability. Common modifications include C-terminal amidation and N-terminal acetylation, which shield the peptide from enzymatic attack [3,56].

Another significant challenge is the poor bioavailability of peptides, particularly when administered orally. Their hydrophilic nature makes it difficult for peptides to cross biological membranes, and they are often degraded in the gastrointestinal tract. To address this limitation, alternative delivery methods, such as non-invasive approaches like nasal administration, are being explored [57]. For example, in a study by de Vries et al., researchers developed an intranasal lipopeptide designed to inhibit viral fusion [58]. Administered via the nasal route, this peptide demonstrated substantial efficacy in preventing the direct-contact transmission of SARS-CoV-2 in ferret models. This approach underscores the potential of nasal delivery systems for peptides, providing a practical solution to enhance their therapeutic potential.

Peptide drug molecules are typically weak immunogens; however, several strategies can be applied during peptide design to reduce their immunogenic risks. Reducing the size of the peptide or mutating non-essential hydrophobic residues, which are more easily recognized by the host immune system, can help to mitigate these risks and improve their therapeutic effectiveness.

In conclusion, while peptide inhibitors offer considerable therapeutic promise, overcoming their limitations through chemical modifications, innovative delivery systems, and advancements in peptide engineering will be essential for enhancing their clinical viability and effectiveness. Beyond peptides, other classes of inhibitors, such as small-molecule inhibitors and monoclonal antibodies, have also been explored for therapeutic purposes.

Despite the challenges associated with peptides, the case study of the *h*ACE2 protein and SARS-CoV-2 spike protein highlights the significant potential of the structure-guided development of peptidic inhibitors targeting protein–protein interactions. This potential has been supported not only by in vitro studies but also by in vivo experiments, as recently presented by Oliveira et al. [59]. The authors identified a wild-type 25-mer mimetic peptide (E25-A46) of hACE2 as a promising drug candidate. In their study, they treated mice, already infected with SARS-CoV-2, via the nasal instillation of the peptide. Administering the peptide 24 h post-infection led to noticeable improvements in clinical symptoms associated with experimental COVID-19, as evidenced by the body weight and clinical scores of the mice. These factors were monitored daily over a five-day period, showing significantly better outcomes in the treated group compared to untreated controls [59].

Overall, both in vitro and in vivo studies support the use of peptides as viable candidates for preventing SARS-CoV-2 infection and mitigating COVID-19-related symptoms.

Peptides are worth considering as a promising alternative to small molecules, such as the recently developed cepharanthine, which may be associated with different side effects [60,61].

Using *h*ACE2 and spike protein interaction as an example, this review demonstrated that PPIs with peptide-based approaches can develop new inhibitors and should be considered as new potential drug targets against many human pathogens, particularly in the era of increasing microbial antibiotic resistance, which poses a high risk of new pandemic episodes [62].

## Figures and Tables

**Table 1:** Interaction interface data.

Feature ^1^	*h*ACE2	Spike
No. of interface residues	19	17
Interface area	825	863
No. of H-bonds	11	
No. of salt bridges	1	
No. nonbonded contacts	106	

^1^ Based no PDBsum data (https://www.ebi.ac.uk/thornton-srv/databases/pdbsum/ accessed on 13 August 2024).

**Table 2:** Sequences of native peptides based on *h*ACE2.

*h*ACE2:		19	20	21	22	23	24	25	26	27	28	29	30	31	32	33	34	35	36	37	38	39	40	41	42	43	44	45	46
First Author	Peptide Name	S	T	I	E	E	Q	A	K	T	F	L	D	K	F	N	H	E	A	E	D	L	F	Y	Q	S	S	L	A
Larue [33]	SAP1																												
	SAP2																												
	SAP5																												
	SAP6																												
Pei [35]	SI4α																												
	SI5α																												
Abouhajar [36]	[30,31,32,33,34,35,36,37,38,39,40,41,42]																												
	[22,23,24,25,26,27,28,29,30,31,32,33,34,35,36,37,38,39,40,41,42,43,44]																												
Chitsike [37]	Pep1																												

**Table 3:** Sequences of mutated peptides based on *h*ACE2.

*h*ACE2:			19	20	21	22	23	24	25	26	27	28	29	30	31	32	33	34	35	36	37	38	39	40	41	42	43	44	45	46		
First Author	Peptide Name		S	T	I	E	E	Q	A	K	T	F	L	D	K	F	N	H	E	A	E	D	L	F	Y	Q	S	S	L	A		
Karoyan [38]	P1																													^(1)^		
	P2			A	L				L								L							L			L			^(1)^		
	P3			A	L				L								L							L			L	A		^(1)^		
	P4	^(2)^		A	L				L								L							L			L	A		^(1)^		
	P5			A	L				L								L						P	L			L	A		^(1)^		
	P6			A	L				L								L							L			L				L	^(1)^
	P7			A	L				Y								L							L			L				L	^(1)^
	P8			A	L				L								M							L			L	A		^(1)^		
	P9			A	L				Y								M							L			L			^(1)^		
	P10			A	L				Y								M							L			L	A		^(1)^		
Chitsike [37]	Pep2																												^(3)^			
	Pep3								V		Y												R	D								
	Pep4										P			E		L	L							L			L	E				
	Pep5										P			E		L	L						C^D^	L			L	E				
Chopra [41]	BP19	^(4)^															I															
Rajpoot [42]	13AApi																		N	F	K											
Odolczyk [43]	pep1c															G																
	pep1d															G																
	pep1e																						G									
	J3														Y	G																

Additional modifications: ^(1)^ -NH_2_; ^(2)^ Ac-; ^(3)^ -GLGKGDFR; C^D^ -D-cysteine; ^(4)^ SLVAVTAAQ-.

**Table 4:** The activity characteristics of the *h*ACE2-derived peptides against *h*ACE2-RBD interaction.

Peptide Name	Modified *	Verification Method	IC_50_ **	Binding Affinity (K_D_) **	Ref.
SAP1		LA (IC_50_); affinity precipitation assays (K_D_)	2.39 ± 0.20 mM	0.53 ± 0.01 mM	[33]
SAP2			3.72 ± 0.37 mM	10.7 ± 4.2 mM	
SAP4			>7.5 mM	-n/d	
SAP5			>7.5 mM	n/d	
SI5α		ELISA	(EC_50_) 1.59 μM	n/d	[35]
[30,31,32,33,34,35,36,37,38,39,40,41,42]		SPR	0.65 μg/mL	n/d	[36]
[22,23,24,25,26,27,28,29,30,31,32,33,34,35,36,37,38,39,40,41,42,43,44]			2.00 μg/mL	n/d	
P8		ELISA (IC_50_); BLI (K_D_)	46 nM	24 ± 11 nM	[38]
P9			53 nM	0.09 ± 0.08 nM	
P10			42 nM	0.03 ± 0.01 nM	
Pep1		AlphaScreen™ assay	80 μM	n/d	[37]
Pep2	Y		113 μM	n/d	
Pep3	Y		72 μM	n/d	
Pep4	Y		42 μM	n/d	
Pep5	Y		363 μM	n/d	
BP19	Y	ELISA	2.08 ± 0.38 μM	n/d	[41]
13AApi	Y	ELISA	>100 μM	n/d	[42]
pep1c	Y	ELISA (IC_50_); MST (K_D_)	n/d	280 ± 60 nM	[27]
pep1d	Y		3.3 mM	210 ± 50 nM	
pep1e	Y		n/d	1900 ± 400 nM	
J3	Y	ELISA (IC_50_); MST (K_D_)	n/d	~50 nM	[43]

* Y—yes; ** n/d—no data.

## Data Availability

All relevant data are presented within the paper.
